# Inhomogeneous ensembles of radical pairs in chemical compasses

**DOI:** 10.1038/srep35443

**Published:** 2016-11-02

**Authors:** Maria Procopio, Thorsten Ritz

**Affiliations:** 1 Department of Physics and Astronomy, University of California, Irvine, 92697-4345, USA

## Abstract

The biophysical basis for the ability of animals to detect the geomagnetic field and to use it for finding directions remains a mystery of sensory biology. One much debated hypothesis suggests that an ensemble of specialized light-induced radical pair reactions can provide the primary signal for a magnetic compass sensor. The question arises what features of such a radical pair ensemble could be optimized by evolution so as to improve the detection of the direction of weak magnetic fields. Here, we focus on the overlooked aspect of the noise arising from inhomogeneity of copies of biomolecules in a realistic biological environment. Such inhomogeneity leads to variations of the radical pair parameters, thereby deteriorating the signal arising from an ensemble and providing a source of noise. We investigate the effect of variations in hyperfine interactions between different copies of simple radical pairs on the directional response of a compass system. We find that the choice of radical pair parameters greatly influences how strongly the directional response of an ensemble is affected by inhomogeneity.

Behavioral experiments suggest that migratory birds and other animals have a physiological magnetic compass sensor that detects the direction of the geomagnetic field^1^,^2^. Two main biophysical mechanisms are currently discussed to underlie magnetic sensing. One is based on mechanical forces on magnetite particles^3^,^4^, the other, explored further here, posits a quantum coherent process involving photoinduced radical pair reactions within photoreceptor proteins with cryptochromes as candidate photo-magnetoreceptors^5^,^6^,^7^. Although the sensory mechanism is still unclear^8^,^9^,^10^, a number of recent studies lend support to the radical pair mechanism^11^,^12^, its physiological basis^13^,^14^,^15^ and the suggested cryptochrome proteins as photo-magnetoreceptors^16^,^17^,^18^,^19^,^20^,^21^, thereby raising the possibility that a chemical sensor could operate in biological systems. Figure 1 illustrates key elements of a radical-pair-based magnetic compass system within a bird. The initial detection of the magnetic field is thought to occur in an ensemble of cryptochromes. At any given time, a subset of all cryptochromes is activated through light, leading to changes in oxidation states in their bound flavin cofactors. An external magnetic field can affect intermediate radical pair states during oxidation or reduction reactions, ultimately leading to changed kinetics in reaching the signaling states of light-activated cryptochromes, thereby influencing the concentration of as of yet unknown downstream signaling partners^7^,^22^,^23^,^24^. The effects of the external magnetic field on any given cryptochrome depend on the intensity as well as the direction of the magnetic field because of the anisotropy of internal magnetic interactions, in particular of hyperfine interactions^5^,^12^,^23^,^25^,^26^,^27^. Assuming an aligned and immobilized ensemble of cryptochromes will lead to directional sensitivity of a receptor cell containing such a cryptochrome ensemble^7^,^22^,^28^,^29^,^30^. The outer segments of photoreceptor cells with their layers of stacked membranes would provide a natural place for such an ensemble, and cryptochromes have indeed been detected in the UV cone photoreceptor of birds^15^,^31^,^32^. Ultimately, the signals from multiple receptor cells would lead to a neuronal representation of the directional magnetic signal.

Regardless of the detailed mechanism of signal transduction, which remains still unknown^7^,^33^, the question arises how one should design a radical pair ensemble so as to optimize its initial directional sensitivity. Beside kinetic considerations, one key factor is the nature of internal magnetic interactions^23^. It has been shown that directional sensitivity is optimized for strong internal magnetic fields, which can be realized by the absence of hyperfine interactions on one of the two radicals^34^,^35^,^36^,^37^. More realistic models have studied how realistic^29^,^38^ and phenomenological^36^,^39^,^40^ spin relaxation mechanisms in radical pairs, and geometrical disorder inherent in biological cells^22^,^28^,^29^ affect the directional response of an ensemble of radical pairs.

So far, all models assume a homogeneous ensemble of radical pairs and consequently that hyperfine interactions are identical for all radical pairs.

However, this assumption is unrealistic because proteins and their attached radical co-factors will be buffeted by the unavoidable molecular motions at physiological temperatures. Based on the tier model of energy landscapes^41^, proteins motions occur over many time scales, and one expects protein fluctuations generally being larger for slower time scales. Such motion on time scales larger than radical pair reaction times will result in varied conformational states even in an ensemble of initially completely identical protein complexes. The protein movement affects quantum states of cofactors, as observed as inhomogeneous broadening of energy lines, e.g. in photosynthetic proteins^42^. Fluctuations of hyperfine interactions in a flavin cofactor were estimated on short time scale^43^, and similarly sized variations are expected to occur on larger time scale as well. Here, we assume a relatively modest level of variation, i.e. 2% of the value of hyperfine interactions.

In addition to the unavoidable inhomogeneities due to molecular motion at physiological temperatures, additional variations of hyperfine couplings may occur if the magnetoreceptor ensemble involves inherently heterogeneous copies. For the candidate photo-magnetoreceptor cryptochrome specifically, such additional inhomogeneity could arise from different dimerization states^44^,^45^,^46^, different states due to rearrangements of the C-terminus of cryptochrome for binding signaling proteins^47^,^48^,^49^, or due to metabolites binding^50^,^51^,^52^, as illustrated in Fig. 1(d).

Variations in hyperfine interactions of the flavin cofactor due to changes in the protein environment, e.g. due to substrate binding, altered geometries of hydrogen bonds, surrounding amino acids have been shown experimentally^53^,^54^,^55^,^56^,^57^,^58^,^59^,^60^,^61^ and theoretically^43^,^62^,^63^. If such inherent heterogeneities existed, they could potentially lead to significantly larger levels of variation than the 2% level assumed here. We therefore also investigate ensembles with larger levels of variations in hyperfine couplings.

By evaluating changes in the directional response of several radical pair magnetic compass systems, we investigate whether some nuclear spin environments lead to directional responses that are more robust to variations of hyperfine interactions than others. We find, interestingly, that some ensembles are dramatically less affected by such hyperfine variations than others. Figure 2 shows an ensemble with interesting, quadrumodal dependence on the geomagnetic field that is, however, a dramatic example of a non-robust system. If one considers variations of hyperfine couplings in the ensemble, the seeming directional sensitivity ends up being buried in the noise. In contrast, one can find ensembles that are much less affected by variations of hyperfine couplings. We find this observation to be true in radical pairs with spin-1/2 nuclei that have been used in many models in the literature as well as radical pairs with spin-1 nuclei, as they occur e.g. in nitrogens of flavin cofactors. Likewise, we see separation into robust and non-robust ensembles in two types of multinuclear spin systems with more realistic hyperfine parameters taken from flavin cofactor and tryptophan radicals. This qualitative conclusion does not depend on the exact size of variations assumed. Certainly, the effects on the magnetoreceptor responses become more pronounced for larger size of variations, but the observation of a robust class of ensembles remains that displays four or more orders of magnitude less signal uncertainty than non-robust cases (cf. Table 1). This significant difference is noteworthy as it might provide an evolutionary target by favoring ensembles that are robust to unavoidable hyperfine coupling variations. Moreover, we find that an intriguing interplay exists between optimal and robust radical pair ensembles and suggests that evolution can select radical pairs that are both optimal and robust.

## Methods

### Theory

A single radical pair comprises a pair of radicals created by photoinduced electron transfer in a spin-correlated singlet or triplet state. In its basic model^5^ each radical has an unpaired electron spin coupled to an external magnetic field via Zeeman interaction and a few local nuclei via hyperfine interactions, described by the following spin Hamiltonian:


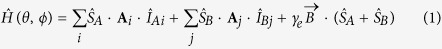


where 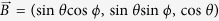 is the external magnetic field, with *θ* and *ϕ* being the polar and azimuth angles, which define the direction of 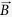 with respect to the radical pair frame. *γ*_*e*_ is the electron gyromagnetic ratio, 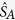, 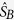 the electron spin operators, 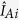 and 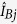 the nuclear spin operators, and **A**_*i*_ and **A**_*j*_ the hyperfine coupling tensors of the radical A and B. Dipolar and exchange interactions are neglected as is typical for most radical pair compass studies (but see ref. 27).

A simplified radical pair reaction model combines coherent quantum spin dynamics of singlet-triplet interconversion, driven by the spin Hamiltonian 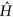, with the kinetics of spin-selective reactions, in which singlet and triplet pairs decay into distinct singlet and triplet reaction products^64^.

Following the Haberkorn approach, we describe spin-selective reactions by using phenomenological rate equations with first-order rate constants *k*_*S*_ and *k*_*T*_ for the singlet and triplet decay^65^. We point out that other approaches based on the theory of quantum measurements are currently under discussion^66^,^67^,^68^,^69^,^70^. The time evolution of a radical pair reaction is thus governed by the following phenomenological master equation:


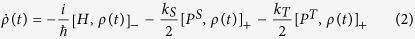


where *P*^*S*^ and *P*^*T*^ are, respectively, the singlet and triplet projection operators. We consider a spin-independent decay rate *k*_*S*_ = *k*_*T*_ = *k*^25^,^71^,^72^, and assume that a radical pair is created in a singlet state, *ρ*(0) = *P*^*S*^/Tr[*P*^*S*^].

The singlet yield Φ, i.e. the amount of products decaying via the singlet channel, is calculated according to 
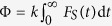
25,73,74, with 
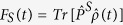
 being the time dependent singlet probability, and results^25^,^71^,^73^:


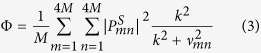


where *ν*_*mn*_ = *ν*_*m*_ − *ν*_*n*_ are energy gap between pairs of eigenstates |*n*〉 and |*m*〉 of the spin Hamiltonian 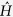. *M* is the number of nuclear spin states and 
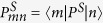
, with *P*^*S*^ being the singlet projection operator. *k* is the spin-independent rate constant capturing all processes leading to a loss of spin correlation. We call *τ* the lifetime of the radical pair, which is given by *τ* = *k*^−1^.

We do not explicitly model spin relaxation mechanisms.

We define the *signal strength*, or maximum angular sensitivity, as ΔΦ = Φ^*max*^(*θ*, *ϕ*) − Φ^*min*^(*θ*, *ϕ*), i.e. the difference between the maximum and minimum value of Φ(*θ*, *ϕ*), and the *anisotropy* as *Λ*(*θ*, *ϕ*) = Φ(*θ*, *ϕ*) − 〈Φ(*θ*, *ϕ*)〉, where 〈Φ(*θ*, *ϕ*)〉 is the spherical average. We represent *Λ*(*θ*, *ϕ*) in polar coordinates, leading to a three-dimensional anisotropic pattern.

### Model

We consider an ensemble of *N* radical pairs arranged to form perfectly ordered crystals with all radicals aligned and immobilized within photoreceptor cells. In a *homogeneous ensemble*, as generally considered previously in the literature, all *N* radical pairs have identical hyperfine tensors {**A**^*n*^} = **A**^*o*^, *n* = 1, *N*, and thus the same singlet yield Φ^*n*^(*θ*, *ϕ*) = Φ^*o*^(*θ*, *ϕ*), and the same anisotropic pattern *Λ*^*o*^(*θ*, *ϕ*), which in turn it represents the directional response of the ensemble. In an *inhomogeneous ensemble*, hyperfine tensors vary between different copies of radical pairs. Lacking detailed information about the type of variations in hyperfine couplings in protein-cofactor systems, we model the variations of *N* hyperfine tensors as being drawn from a normal distribution around **A**^*o*^. In particular, for each hyperfine interaction included in the model, we draw a Gaussian distribution 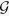 for each hyperfine tensor component 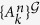, around the component values 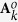, *k* = *x*, *y*, *z*. We call Δ the standard deviation of a Gaussian distribution. We compute *N* singlet yields Φ^*n*^(*θ*, *ϕ*) and anisotropic patterns *Λ*^*n*^(*θ*, *ϕ*), corresponding to the *N* normally distributed hyperfine tensors. The directional response of the inhomogeneous ensemble is the average singlet yield 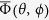, obtained by averaging over the *N* individual singlet yields, with a corresponding average pattern 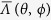. The signal strength of the inhomogeneous ensemble is obtained as 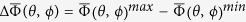. We call the standard deviation of the average singlet yield, *σ*(*θ*, *ϕ*), *signal uncertainty*, whose average over all directions 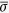 is used to evaluate the overall uncertainty of the directional response of the inhomogeneous ensemble. We define the signal-to-noise ratio as S/N, and consider the signal S being the signal strength 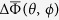 and the noise N being the signal uncertainty *σ*(*θ*, *ϕ*).

### Estimates of hyperfine coupling variations

It is not yet possible to perform quantum mechanics-molecular mechanics (QM-MM) simulations on a 100 microsecond - 1 millisecond timescale so as to evaluate the effect of conformational changes on these timescales on hyperfine couplings of flavin cofactors in cryptochromes. Such calculations are furthermore complicated by the absence of detailed structural information of the active form of cryptochrome. Finally, experimental values of hyperfine interaction variations for the desired protein systems are not readily available. We therefore base our estimate on two different approaches.

First, we can compare hyperfine coupling values between copies of a flavin radical of the same protein when substrates are added through experimental measurements. While such measurements do not exist for cryptochromes from birds, we can use values available for other flavoproteins.

The spin density of the ring system (i.e. isoalloxazine moiety) of a flavin cofactor radical of flavoproteins, including cryptochromes^61^, is highly sensitive to change in the protein environment. This has allowed hyperfine spectroscopy methods to use flavin cofactor radicals as a spin probe^59^,^60^,^61^,^62^,^63^. Particularly, electron nuclear double resonance (ENDOR) studies of flavoproteins have shown changes of proton hyperfine couplings of the flavin’s isoalloxazine moiety upon substrate binding^53^,^54^,^56^. The measured proton hyperfine couplings changed on average by about 2%^56^, 5%^54^ and 10%^53^ in different flavoproteins. The 2% change was for enzyme-substrate binding in Escherichia coli DNA photolyase, which is highly homologous to cryptochrome^75^.

Secondly, through QM-MM simulations hyperfine interactions of flavin radicals were estimated to fluctuate on short time scales (picoseconds) with an average magnitude of 0.06 mT^43^. The smallest magnitude was 0.02 mT while the largest 0.1 mT. These short fluctuations would be averaged out in a radical-pair-based compass magnetoreception^38^, but we use them as a lower-bound estimate for the size of the relevant long time fluctuations that are beyond the reach of QM-MM calculations.

Based on such estimates, we model variations of hyperfine interactions with Gaussian distributions and consider a percentage-based standard deviation of 2% and a fixed-width standard deviation of 0.06 mT.

In the following, we show the results for a Gaussian with a width of 2% of the initial hyperfine components 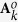, *k* = *x*, *y*, *z*. We have performed additional calculations with percentage values of 5% and 10%, as well as with fixed widths of 0.02 and 0.1 mT for all components and find the results to be qualitatively unchanged (see Tangle 1, Table 2). The only exception occurs for radical pairs with the limiting axiality value of *α* = 1, which will be discussed in more detail below (see Table 1).

## Results

Figure 2 depicts the anisotropic pattern, signal strength, and signal uncertainty of a particular inhomogeneous ensemble of radical pair reactions and represents the rationale for this study. For comparison, Fig. 2(a) depicts an intriguing anisotropic pattern *Λ*^*o*^ with quadrumodal directional sensitivity for a homogeneous ensemble of *N* radical pairs with identical hyperfine tensors. Figure 2(b) shows a section (azimuth angle *ϕ* = 0) of the corresponding singlet yield as a function of the polar angle *θ*, indicating a sizeable signal strength. When considering inhomogeneity, as expected in realistic biological environments, variations of hyperfine tensors lead to different and complex anisotropic patterns for each radical pair as illustrated in Fig. 2(c). The directional information from the inhomogeneous ensemble stems from the average of the singlet yields, shown (for *ϕ* = 0) in (e) and as a three-dimensional anisotropic pattern in (d). It is noteworthy that the average pattern has qualitatively different features from the homogeneous pattern, as the quadrumodal character is now averaged out. More importantly, there remains a significant random component, uncertainty in the signal, as illustrated in both Fig. 2(d,e) by showing the average pattern together with one standard deviation *σ* of the signal variation. In the particular radical pair ensemble chosen here, the signal uncertainty *σ* is of comparable size to the maximum angular sensitivity or signal strength 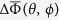. Clearly, in order to achieve a large signal-to-noise ratio, a reliable compass sensor not only needs to maximize the signal strength, but also needs to minimize the signal uncertainty.

In the following, we ask whether there are choices of hyperfine values that reduce the signal uncertainty while also resulting in large signal strengths, thus leading to a robust and optimal compass sensitivity. To evaluate the robustness of the whole signal we use the average signal uncertainty 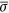, obtained by averaging *σ*(*θ*, *ϕ*) over all directions. We study simple radical pair models with up to two hyperfine interactions and only axial anisotropy, thereby limiting the parameter space and computational cost, and vary hyperfine parameters in a range of values typically encountered in organic radicals. We also investigate the effects of inhomogeneity between radical pair copies in more realistic chemical compasses by studying two types of multinuclear radical pair systems with realistic hyperfine interactions taken from flavin and tryptophan radicals^30^. Moreover, in all calculations we use radical pair lifetimes of the order of microseconds as suggested by theoretical interpretations of experimental results^38^. In particular, we report results of the simple radical pair models with a lifetime of *τ* = 10 *μ*s in Figs 3, 4 and 5, and of the more realistic models with a lifetime of *τ* = 1 *μ*s in Fig. 6.

### One-HFI radical pair models

First, we consider the simplest radical pair model that can account for directional magnetic field effects, in which only one of the two electron spins is coupled to a nucleus through an axial hyperfine interaction, whereas the other radical is devoid of hyperfine interactions. We consider a spin-1/2 nucleus as in hydrogen. This H(ydrogen)-model has been extensively studied as a proof of principle in the literature^25^,^36^,^39^,^73^. We also consider a N(itrogen)-model with a spin-1 nucleus, motivated by the fact that the largest hyperfine coupling in cryptochromes is of this type. The Hamiltonian in Eq. 1 for these models has *i* = 1, *j* = 0, and the hyperfine tensor is axial. We parametrize the axial symmetry as follows: *A*_*x*_ = *A*_*y*_ = *a* − *aα* and *A*_*z*_ = *a* + 2*aα* with *a* being the strength and *α* the axiality of the hyperfine interaction. The axial symmetry allows us to set the azimuth angle *ϕ* = 0, without loss of generality. We consider an ensemble of *N* = 3000 inhomogeneous copies of radical pairs, where each radical pair has hyperfine tensor components drawn from Gaussian distributions with averages *A*_*x*_ = *A*_*y*_ = *a*(1 − *α*) and *A*_*z*_ = *a*(1 + 2*α*) and standard deviations being the 2% of respectively *A*_*x*_ and *A*_*z*_. We compute the signal strength 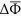 and signal uncertainty 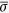 of the inhomogeneous for average hyperfine coupling strengths *a* between 0.05 mT and 5 mT, and all possible values for the axiality *α*. The strength of the external magnetic field is set to *B* = 0.05 mT.

The signal strength 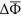 as a function of *a* and *α*, and for a lifetime *τ* = 10 *μ*s, is depicted in Fig. 3(a) for the H-model and (c) for the N-model. The two models show similar features, and we can identify three regions. The optimal region of maximum signal strength occurs for axiality *α* = 1, with a signal strength of 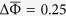, for *a* ≫ 0.5 mT, for the H-model and 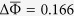, for *a* ≫ 0.2 mT, for the N-model, both in agreement with previous studies on a single radical pair reaction^36^,^37^. For the H-model, a large plateau region, depicted in green, with a constant signal strength of 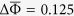, can be observed for sufficiently large hyperfine strengths (*a* ≫ 0.5 mT) and away from the special axiality values *α* ≠ −2, 0, 1. A similar plateau region is harder to identify for the N-model, but may occur with a less pronounced transition in signal strength and a smaller constant signal strength of 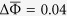, or *a* ≫ 0.15 mT and −1.0 < *α* < 0 (within the blue area). Outside of the maximum and plateau regions, the signal strength depends strongly on the axiality. In general, larger axiality enhances the signal strength, while smaller axiality reduces signal strength.

Figure 3 shows the signal uncertainty 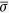, in logarithmic scale, as a function of the hyperfine parameters for (b) the H-model and (d) the N-model, respectively.

For the H-model, an increase in signal strength appears to correlate with a decrease in signal uncertainty at first sight, as indicated by the similarity in qualitative features in Fig. 3(a,b). However, when plotting a histogram of signal uncertainty 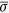 versus signal strength values 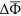 (see Fig. 4(a)), we find that there is no simple correlation between signal strength and signal uncertainty. Rather, a strongly diminished signal uncertainty can be observed for two specific values of signal strength, namely 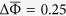 and 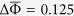, i.e. the optimal signal strength and the signal strength in the plateau region, respectively. Similarly, in the N-model, we find robust regions of greatly suppressed noise - or signal uncertainty - to occur at the two signal strength values of 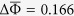, the optimal value, and of 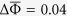 (see Fig. 4(d)). The region with 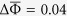 corresponds to values of −1.0 < *α* < 0 and *a* ≫ 0.15 mT in the tentative labeled plateau region in Fig. 3(c,d).

Keeping a lifetime of *τ* = 10 *μ*s, we repeated the calculations with 5% and 10% variations, and observed qualitatively the same results. That is, we could identify in every case a plateau and optimal region in signal strength of the same size as with the 2%, i.e. 0.125 and 0.25 for the H-model and 0.04 and 0.166 for the N-model, and found that both of these regions coincided with a dramatic decrease in signal uncertainty. Table 1 reports the signal strength 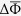, signal uncertainty in logarithm scale 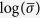, and signal-to-noise ratio S/N of selected radical pairs for different percentage-based standard deviations Δ, for (a) the H-model and (b) the N-model. The selected radical pairs are representative for the optimal, plateau and least robust regions. Interestingly, in all calculations (see Fig. 4(a,d) and Table 1) we find that, while the signal strength varies only within a comparatively small range, the signal uncertainty changes by four to five orders of magnitude for different selections of hyperfine parameters. This difference is noteworthy because leads to radical pairs in the optimal and plateau regions with a signal-to-noise ratio of at least four order of magnitude larger than radical pairs in the least robust region (see Table 1).

Moreover, we repeated the calculations with 2% variations for lifetimes of 1 *μ*s and 100 *μ*s, and find the same qualitative results as with a lifetime of 10 *μ*s. If the lifetime is decreased to 0.1 *μ*s, we begin to see a reduction of signal strength. For the H-model, such a reduction in signal strength as a function of the rate constant can be analytically predicted. Indeed, the signal strength values in the plateau and optimal regions can be determined analytically in the limit of sufficiently large hyperfine interaction *a* ≫ *ω* and sufficiently slow rate constant *a* ≫ *k*, where *ω* = *γ*_*e*_*B*. The signal strength for the plateau region (*α* ≠ −2, 0, 1) is 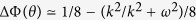, and for the optimal region (*α* = 1) is 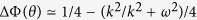, which reduces, respectively, to 0.125 and 0.25 for *a* ≫ *ω* ≫ *k*, i.e. when the rate constant is slow enough to allow the geomagnetic field to affect the spin dynamics^76^.

### The optimal case of *α* = 1

As mentioned above and in ref. 36, a one-HFI radical pair shows the maximal signal strength for the unique case of an anisotropy *α* = 1, i.e. when two components of the hyperfine tensor in its diagonal form satisfy *A*_*x*_ = *A*_*y*_ = 0. Regardless of whether this case can be actually realized in a biological cofactor, one can ask whether this optimal case also has a desirable low signal uncertainty. We find that the answer to this question depends on the details of the distribution of hyperfine couplings for inhomogeneous ensembles. If we assume that variations will vary by 2%, 5% and 10% of the initial hyperfine value of each component, then such variations will leave the lateral components unchanged at zero, *A*_*x*_ = *A*_*y*_ = 0. In this case, the optimal case is also robust, because is unaffected by inhomogeneous noise, leading to minimal signal uncertainty and maximum signal-to-noise ratio as seen in Figs 3(b,d) and 4(a,d) and in Table 1.

However, there is no a priori reason why hyperfine couplings should not also vary in x and y directions and using a percentage-based variation may therefore lead to an underestimation of inhomogeneous noise in the case where the hyperfine coupling (or a component of it) is very small to begin with. To model an alternative distribution, we considered one-HFI models with hyperfine couplings drawn from a Gaussian distribution with a constant (i.e. 0.02, 0.06 and 0.1 mT), rather than percentage-based standard deviation for all hyperfine tensor components. Table 1 summarizes the results of the one-HFI models for a percentage-based and a fixed-width standard deviation. We find that inhomogeneity has two effects on a radical pair ensemble centered around hyperfine interactions with *α* = 1 (i.e. optimal region). It reduces the signal strength, because the variations cause the hyperfine couplings to deviate from the optimal anisotropy of *α* = 1. In addition, it increases the signal uncertainty, and consequently reduces the signal-to-noise ratio. For example, comparing the 5% with the 0.06 variation, the signal strength in the H-model reduces to 0.2 and the signal uncertainty increases from 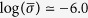 to 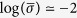, with a concomitant reduction of about four order of magnitude signal-to-noise ratio. For larger variations such a loss of robustness can increase signal uncertainty until it becomes comparable in size to the signal strength. It is noteworthy that the plateau regions discussed above are insensitive to the exact form of hyperfine variations. Changing from a percentage-based to a constant standard deviation in the distribution has no effect on either signal strength and signal uncertainty, as long as the magnitude of the standard deviations is comparable (see Table 1). Both numerical and analytical evaluations bear out that the plateau regions with signal strength of 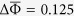 and 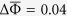 in the H-model and N-model, respectively, correspond to the minimal signal uncertainty, regardless of the details of hyperfine coupling variations. This “robustness” appears to be a special feature of the plateau regions in the one-HFI models. For non robust radical pairs, changing from a percentage-based to a constant standard deviation further increases the signal uncertainty, which in some cases becomes larger than the signal strength. These results suggest that selection of noise-suppressing radical pairs can be an evolutionary target for compass sensors.

### Two-HFIs radical pair models

We extend our study to the slightly more complex situation in which we consider two axial hyperfine interactions. We study two arrangements. In one arrangement, one radical has two hyperfine interactions while the other is devoid of hyperfine interactions, and the Hamiltonian is given by Eq. 1 with i = 2 and *j* = 0. We denote the model with nuclear spin-1/2, 2H-0 model, and with nuclear spin-1, 2N-0 model. In the other arrangement, each radical has one hyperfine interaction and the Hamiltonian is given by Eq. 1 with *i* = 1 and *j* = 1, and we denote the model with nuclear spin-1/2, H-H model, and with nuclear spin-1, N-N model. In all cases, we parametrize the axial symmetry of the hyperfine interactions as with the one-HFI models, i.e. *A*_*x*_ = *A*_*y*_ = *a* − *aα* and *A*_*z*_ = *a* + 2*aα* with *a* being the strength and *α* the axiality. Moreover, we define *ψ* as the polar angle (which is not shown in Eq. 1) between the two hyperfine tensors **A**_1_ and **A**_2_, and without loss of generality we consider the azimuth angle *ϕ* = 0.

We randomly generate 10^5^ samples of nuclear spin environments with average hyperfine strengths, *a*_1_, *a*_2_, ranging from 0.05 to 5 mT, axiality *α*_1_, *α*_2_ from −2 to 2, and the angle *ψ* between the two hyperfine tensors from 0° to 90°. As with the one-HFI models, for each nuclear spin environment we consider an ensemble of 3000 radical pairs, where each radical pair has two hyperfine tensors drawn from Gaussian distributions with average *A*_*xm*_ = *A*_*ym*_ = *a*_*m*_(1 − *α*_*m*_) and *A*_*zm*_ = *a*_*m*_(1 + 2*α*_*m*_), *m* = 1, 2. Using a lifetime of *τ* = 10 *μ*s and a magnetic field strength of *B* = 0.05 mT, we calculate the average signal strength 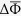 and signal uncertainty 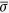 for each nuclear spin environment and for all models. We depict the results of the effects of variations of hyperfine interactions with magnitude 2%.

Figure 4 shows the signal uncertainty 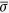 in logarithmic scale as a function of the average signal strength 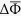 for the (a) H-model, (b) 2H-0 model, (c) H-H model, (d) N-model, (e) 2N-0 model and (f) N-N model. The relationship between signal strength and signal uncertainty for the one-HFI models has been discussed above. Figure 4 depicts how this relationship is affected when another hyperfine interaction is added to the one-HFI models. We note that a crucial factor for both signal strength and signal uncertainty is whether or not one radical remains devoid of hyperfine interactions. For the 2H-0 model (see Fig. 4(b)), the qualitative features resemble those of the H-model (see Fig. 4(a)) with the most robust compass sensors being for signal strengths of 0.125 and 0.25. The signal uncertainty for these values in the 2H-0 model is slightly higher than in the H-model, albeit still much lower than at other signal strength values. Furthermore, the signal uncertainty is slightly reduced for most other values of signal strengths. The trade-off is that it appears harder to realize specialized cases with extremely low signal uncertainty and optimal signal strength in such radical pairs. The features of the 2N-0 model (see Fig. 4(e)) confirm the trends seen in the 2H-0 model, but also show some new features. Regions of very low signal uncertainty can barely be discerned at signal strength of 0.04 (plateau region) and of 0.22 (optimal region), but similarly low uncertainty regions occur for other signal strengths as well. Overall, signal uncertainty is reduced again for most values of signal strengths, trading off with an increase in signal uncertainty for the extremely low uncertainty regions. We also note that two nitrogen nuclei on the same radicals give rise to a compass sensor with a larger directional sensitivity than the one with only one nitrogen, in agreement with a previous study^37^. Analyzing the parameters of radical pairs with large signal strengths for models with two hyperfine interactions on the same radical, we find that optimal and robust sensitivity can be realized when a nuclear spin environment comprises two hyperfine interactions of average strengths *a*_1_, *a*_2_ ≫ 0.05 mT, both with a strong anisotropy 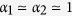, and nearly parallel axes 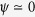. In both models the robust sensitivity of 0.125 and 0.04 is realized when one radical has one hyperfine interaction in the plateau region of the one-HFI models, and the other has a much smaller hyperfine strength.

We repeated the calculations with a lifetime of *τ* = 10 *μ*s and with larger variation of 5% and 10%, and find similar features as with the 2% variation. Table 2 reports the results of selected radical pairs from the optimal, plateau and least robust regions for the different percentage-based standard deviations Δ. The optimal and plateau regions remain the most robust with unchanged signal strength and signal uncertainty that slightly decreases for larger variations. Similarly to the one-HFI model, the noise-suppressing 2HFI-0 model radical pairs have the advantage of a signal-to-noise ratio of at least three order of magnitude larger than non robust radical pairs.

Differing from the one-HFI models (see Tables 1 and 2), when changing from a proportional to a constant standard deviation in the Gaussian distribution we observe similar features as in the proportional case. Although the signal uncertainty slightly decreases, we can find regions that combine large signal strengths (0.125, and 0.25) with extremely low signal uncertainty (see Table 2). As in the proportional case, such optimal and robust compass sensors can be realized with hyperfine axiality close or equal to one, i.e. 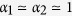. The signal uncertainty of the plateau region slightly decreases as well when changing from a proportional to a constant standard deviation, and the least robust region has at least two order of magnitude less signal-to-noise ratio than optimal and plateau regions.

When both radicals have one hyperfine interaction each as for the H-H and N-N models (see Fig. 4(c,f), respectively), the situation changes. Directional sensitivity is generally reduced. Regions of low signal uncertainty appear to correlate with regions of low signal strength, but clearly such radical pairs do not result in a useful compass sensors.

The fact that there are much fewer hyperfine parameters for the H-H and N-N models that result in combined large signal strength and very low signal uncertainty, indicates that these models are much less suitable for a compass sensor. One exception appears to occur in the N-N model at a signal strength of about 0.11 that shows a signal uncertainty of 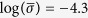. Such exception remains for larger variations of 5% and 10%, where the signal strength remains unchanged to 0.11, and the signal uncertainty resulted, respectively, 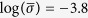, and 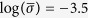. Such radical pair is obtained with two hyperfine strengths much larger that 0.05 mT, axiality close to one, and parallel axis (e.g. *a*_1_ = 0.9 mT, *a*_2_ = 4.3 mT, *α*_1_ = 0.9, *α*_2_ = 1.0 and *ψ* = 10.0). Noteworthy, such a radical pair shows robust feature when changing to a fixed-width standard deviation. For a standard deviation of 0.02 mT, the signal uncertainty resulted 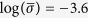, for 0.06 mT 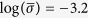, and for 0.1 mT 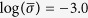.

We also repeated the set of calculations with 2% variation for the following lifetimes *τ* = 0.1, 1, 100 *μs*, and obtained qualitatively analogous results.

### Inhomogeneous vs. homogeneous ensemble

The uncertainty of the signal due to inhomogeneity provides a fundamental problem and the main purpose of our study is to investigate how it could be overcome in building a reliable magnetic compass sensor. However, it is worth pointing out that considering inhomogeneity in a realistic biological ensemble also raises a serious caveat for interpreting patterns of directional sensitivity based on a single set of hyperfine parameters alone, as was routinely done in the literature. This caveat is illustrated already in Fig. 2. The constant set of hyperfine parameters, neglecting inhomogeneity, indicates a quadrumodal directional signal of the magnetic compass with similar singlet yields in four directions, as shown in Fig. 2(a). However, when considering inhomogeneity, the average singlet yield of the inhomogeneous ensemble, shown in Fig. 2(d) displays no more quadrumodal character, but shows a bimodal pattern. Thus, any conclusions about e.g. behavior of an animal based on the homogeneous singlet yield would be invalid, at least for the currently used estimate for the variation strength of hyperfine interactions. Figure 2 shows an example with a particularly high signal uncertainty of 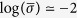.

We now ask how such deviation of the average singlet yield 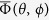 from the yield of the homogeneous ensemble Φ^*o*^(*θ*, *ϕ*) correlates with the signal uncertainty. We quantify the deviation between average and homogeneous singlet yield as 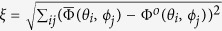. Plotting the signal uncertainty 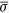 versus the deviation between average and homogeneous pattern *ξ* in Fig. 5, we find a near linear correlation in a double logarithmic scale up to log(*ξ*) = −2. Figure 5 uses 10^5^ ensembles of the H-model with different hyperfine parameters and 3000 inhomogeneous copies for each ensemble, as above. Similar results are found for the other models. Thus we find that for a large class of radical pair models, neglecting variation of hyperfine interactions may be rather misleading, because, when including such variations, the average pattern will differ significantly from the pattern of the homogeneous ensemble. The exception to this caveat occurs when a robust radical pair ensemble with low signal uncertainty is selected. In this case, the pattern does not change noticeably from copy to copy and one can well approximate it with the pattern of a homogeneous ensemble. This robust pattern has a bimodal form with a yield approximating Φ ≈ (1 + cos 2*θ*) in the limit of *a* ≫ *ω* ≫ *k*.

## Multinuclear Radical Pair Systems

The one-HFI and two-HFIs radical pair models, despite being simplified models of realistic chemical compasses, have allowed to explore many nuclear spin environments for finding optimal and robust radical pair parameters. It is computationally prohibitively expensive to perform the same exhaustive study on more realistic radical pairs, with a larger number of hyperfine interactions. To limit the parameter space, we choose therefore to study the effects of inhomogeneity on two types of multinuclear radical pairs with eight hyperfine interactions and realistic hyperfine values of flavin and tryptophan radicals obtained from DFT calculations^30^.

One type of eight-nuclei radical pair system comprises the hyperfine values of two nitrogen nuclei (N5 and N10) of the flavin adenine dinucleotide radical (FADH^•^), and of six hydrogen nuclei (H1, H2, H4, H5, H6, H7) of the tryptophan radical (Trp). For simplicity we call this type FADH^•^-Trp. The Hamiltonian for FADH^•^-Trp is given by Eq. 1 with *i* = 2, and *j* = 6. The other type of radical pair system comprises the hyperfine values of two nitrogen nuclei (N5 and N10) and six hydrogen nuclei (H5, H6, H8*α*, H8*α*, H1′, H1′) of the flavin radical, while the other radical is supposed to be devoid of hyperfine interactions. We call this type FADH^•^-0. The Hamiltonian for FADH^•^-0 is given by Eq. 1 with *i* = 8, and *j* = 0. These two types of radical pair systems can be seen as an extension of the 1HFI-1HFI and 2HFIs-0 models, previously studied, when more nuclei are added to, respectively, both radicals or to only one radical and thereby allow comparisons with previous results. The choice of the nitrogen nuclei arises from studies that shown N5 and N10 being the predominant nuclei in flavin^37^, while hydrogen nuclei are chosen based on their strength.

To model variations of hyperfine interactions we use the method described in the Methods section. We consider the hyperfine tensor components taken from flavin and tryptophan radicals^30^ as average values of Gaussian distributions with fixed-width of 0.06 mT. We choose a fixed-width of 0.06 G because this is the average size of variations of many hyperfine tensors in a flavin radical available from the literature^43^. For each hyperfine tensor component we generate 3000 samples of normally distributed values, and then compute 3000 singlet yields of each eight-nuclei radical pair system. We set the lifetime of the two types of radical pairs to *τ* = 1 *μ*s, and the external magnetic field to *B* = 0.05 mT.

Figure 6 reports (a) and (d) the homogeneous pattern Λ^*o*^, (b) and (e) the average pattern 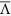, and (c) and (f) several individual patterns Λ^*n*^ for (top) FADH^•^-Trp, and (bottom) FADH^•^-0. The deterioration of the signal due to inhomogeneous noise, or signal uncertainty *σ*, is represented by the grey grid in Fig. 6(b,e). The average uncertainty resulted 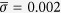 for FADH^•^-Trp, and almost one order of magnitude less, i.e. 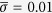, for FADH^•^-0. Both values are within the range of uncertainties of the simplified models (Fig. 4). These results show that the size of the signal uncertainty is affected by the choice of hyperfine parameters even in more extended hyperfine coupling systems, and indicate that multinuclear spin systems are not always more robust to the deteriorating effects of the inhomogeneous noise. Furthermore, the fact that FADH^•^-Trp is more robust than FADH^•^-0 is in agreement with our previous results (see Fig. 4), that show that radical pairs with hyperfine interactions in both radicals are generally more robust to inhomogeneous noise.

Interestingly, the noise affects the shape of the anisotropy differently in the two systems. While in FADH^•^-Trp inhomogeneity changes the shape of (b) the average pattern and (c) the individual patterns with respect to (a) the homogeneous pattern, in FADH^•^-0 the shape of (e) the average and (f) individual patterns remains unchanged with respect to (d) the homogeneous pattern. The shape of the anisotropy is affected by inhomogeneous noise because of a loss of axial symmetry in one radical (given by N5 and N10) when hyperfine interactions are added on the other radical. This is further confirmed by the complex patterns depicted in Fig. 2 where the radical pair system lacks of axial symmetry. Conversely, in all axial systems studied in the one-HFI and two-HFIs models, the shape remains unaffected by inhomogeneous noise and all patterns, i.e. homogeneous, average and individuals, have similar shape to those of Fig. 6 (bottom).

Moreover, the average signal strength is larger in FADH^•^-0 (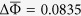) than in FADH^•^-Trp (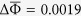), as expected from previous studies^37^. While FADH^•^-0 results more directional sensitive, it is also less robust to inhomogeneous noise than FADH^•^-Trp. The larger signal strength of FADH^•^-0 compensates its smaller robustness, thereby resulting with a signal-to-noise ratio larger by a factor of ten than FADH^•^-Trp.

We repeated the calculations only for the maximum and minimum singlet yield with different fixed-width and percentage based variations, and calculated the signal strength 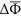, the signal uncertainty 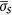 and signal-to-noise ratio, for both models. Table 3 reports the results, and we note that signal uncertainty slightly decreases for larger variations, and reduces when changing to a percentage-based variations. Regardless of the form and magnitude of variations the signal uncertainty of FADH-0 is about ten fold larger than FADH-Tr. However, because FADH-0 has a larger signal strength, the signal-to-noise ratio of FADH-0 results ten fold larger than FADH-Tr.

## Discussion

We have investigated the effects of variations of hyperfine interactions on the directional response of an inhomogeneous ensemble of radical pairs. Evaluating these effects on simple radical pair models, up to two axial hyperfine interactions, each with a wide parameter range, reveals that the nuclear spin environment has a significant influence on the effects of such variations. In fact, some nuclear spin environments can make a radical pair-based compass virtually immune to the influence of variations of hyperfine interactions. Such robustness is a design feature distinct from optimality. An optimal compass sensor can be found when the compass responds to the external magnetic field with a maximum directional sensitivity, whereas a robust sensor is one where the directional response remains unaffected by variations regardless of the magnitude of the directional sensitivity. Both features ultimately affect the signal-to-noise ratio of a magnetic sensory system. The strength of the directional sensitivity can be thought of as the signal and the change of the signal due to variations between copies in the ensemble can be thought of as a source of noise.

Intriguingly, the noise stemming from inhomogeneity is inextricably linked with the choice of a particular radical pair. In other words, evolution cannot change the signal of a radical pair independently from changing the robustness to this source of noise because selection of radical pair parameters will affect both signal and noise robustness at the same time.

The question arises whether it is possible to find radical pairs that are both optimal (i.e. show a maximum signal strength) and robust (i.e. minimize the signal uncertainty) at the same time. For the one-HFI models studied here, the optimal radical pairs are realized for axiality *α* = 1, i.e. all but one component of the hyperfine tensor, when expressed in its principal axis, equals zero. We find that this radical pair is robust to variations of the hyperfine interaction along its primary axis, but not robust to variations in directions perpendicular to it. Assuming that it is possible to realize the theoretical limiting case of *α* = 1, it would thus also be necessary to shield this radical pair from variations perpendicular to its primary axis so as to maximize the signal-to-noise ratio. By sacrificing a relatively modest amount of signal strength (e.g. a factor of two compared to the *α* = 1 for the one-Hydrogen model), one can identify a large robust region where the directional sensitivity is independent of the hyperfine parameters. Such robustness is a general feature for one-HFI radical pairs and can be understood through analytical calculations.

The relationship between optimality and robustness becomes, unsurprisingly, more complex for radical pairs with two hyperfine interactions. In keeping with earlier observations^37^, we find that the response is stronger if one radical is devoid of hyperfine interactions (2HFI-0 model). Moreover, it is more common to find robust radical pairs with large directional sensitivities in 2HFI-0 radical pairs than in 1HFI-1HFI radical pairs. However, even in the less ideal classes, e.g. in the N-N radical pairs, one can still find a sizable group of radical pairs with a directional response larger than 0.1 that are robust, providing no fundamental obstacles for evolutionary selection of radical pairs with favorable signal-to-noise ratios.

Surprisingly, more realistic multinuclear spin systems are not naturally more robust to inhomogeneous noise, and again the choice of hyperfine parameters affects both signal and robustness to inhomogeneous noise at the same time. Indeed, we find that one type of eight-nuclei radical pair system (FADH^•^-0) has a larger directional sensitivity but smaller robustness (of ten fold) than the other type (FADH^•^-Trp) of eight-nuclei system studied here. Overall the signal-to-noise ratio was larger in FADH^•^-0 than in FADH^•^-Trp.

**Figure 1:**
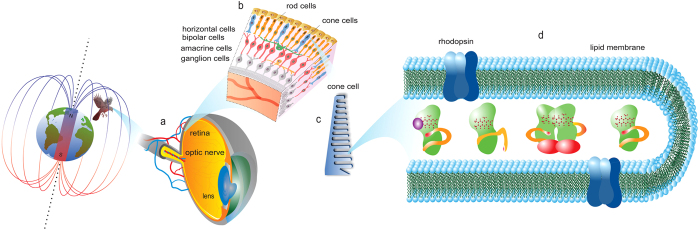
Schematic illustration of the components of a magnetic compass in a bird’s eye. (**a**) The retina is a light-sensitive layer at the back of the eye which converts light signals, entering the eye from the lens, into electro-chemical signals and transmits these signals to the brain via the optic nerve. (**b**) Section of the retina showing the five types of cells arranged in layers. The primary signal is generated in the photoreceptor cone cells, passed to other cell layers, and transmitted to the brain by the ganglion cells. (**c**) Disc of the outer segments of a cone cell where the photo-magnetoreceptor cryptochrome is suggested to be localized^15^,^32^. (**d**) Section of a disc membrane of a cone cell. Rhodopsin proteins, depicted in blue, perform the primary photo-transduction of visual information. Cryptochrome proteins, depicted in green with the C-terminus in orange, may perform the primary transduction of magnetic information, via formation of magnetically sensitive radical pair reactions. An ensemble of cryptochromes within photoreceptor cells can be in different conformational states such as (from left to right): with the C-terminus in proximity to the flavin cofactor, with a metabolite bound, in dimerized form, with the C-terminus extended. “Copyright (c) 2015 Maria Procopio and its licensors. All rights reserved.”

**Figure 2:**
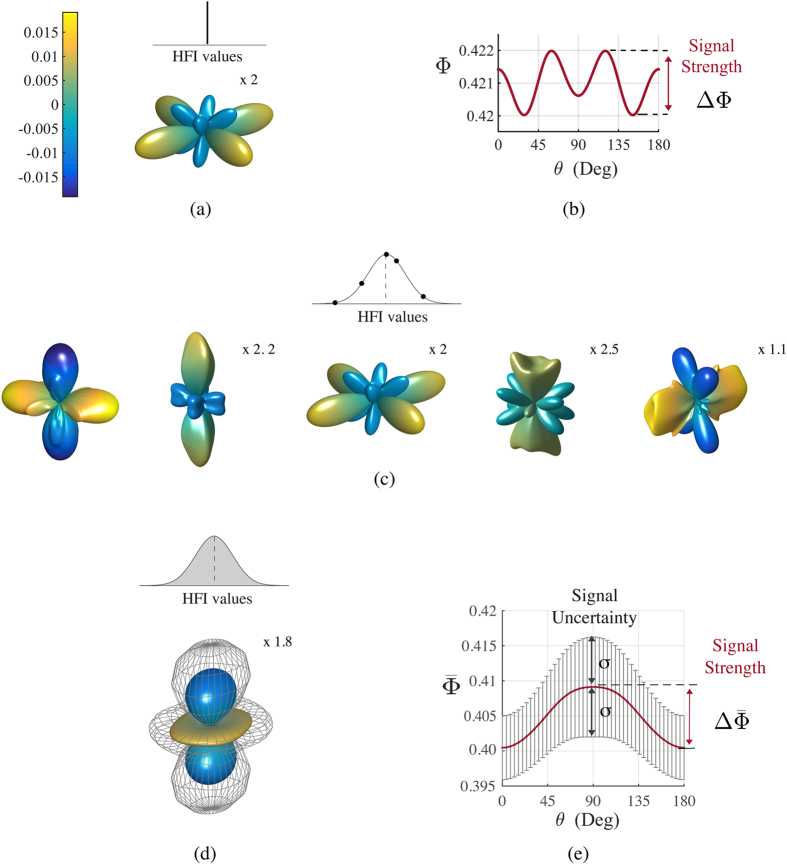
Dramatic example of the anisotropic response of a particular homogeneous ensemble of *N* radical pairs, compared with the anisotropic response of the ensemble when inhomogeneity is considered. In a homogeneous ensemble *N* copies of radical pairs have identical hyperfine values **A**^*n*^ = **A**^*o*^, thus identical singlet yields Φ^*n*^ = Φ^*o*^ and anisotropic patterns *Λ*^*n*^ = *Λ*^*o*^. When considering inhomogeneity between radical pair copies the *N* hyperfine values vary between radical pairs. We model variations of hyperfine interactions with Gaussian distributions 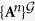 around **A**^*o*^. (**a**) Anisotropic response of the homogeneous ensemble *Λ*^*o*^, and (**b**) singlet yield Φ^*o*^ for *ϕ* = 0. The signal strength is given by ΔΦ^*o*^, i.e. the difference between the maximum and minimum values of Φ^*o*^. (**c**) Complex anisotropic patterns *Λ*^*n*^ for specific hyperfine parameters of different radical pairs of the inhomogeneous ensemble. (**d**) Average anisotropic response of the inhomogeneous 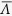, obtained by averaging over all *N* anisotropic yields Φ^*n*^. Noise due to inhomogeneity leads to a signal uncertainty *σ* (or standard deviation), represented as a grey grid. (**e**) Section of the average singlet yield 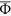 for *ϕ* = 0, with average signal strength 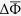 and uncertainty represented by *σ*. The average signal uncertainty (not shown here) is 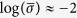 (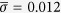). The number of radical pairs is *N* = 3000. Blue and yellow colors represent singlet yield values, respectively, larger and smaller than the spherical average. The radical pair model is the N-N model, where each radical is coupled to a nitrogen nucleus. The two hyperfine values are the same, correspond to N5 in flavin^30^, and are arranged perpendicularly. The hyperfine tensors components used for N5, in its principal axis, are *A*_*x*_ = −104.9, *A*_*y*_ = −99.6, *A*_*z*_ = 1382.6 in *μ*T (for details see ref. 30). The magnitude of variations is 5%, the lifetime of the radical pair is *τ* = 0.1 *μ*s and the strength of the external magnetic field is *B* = 0.05 mT. The color bar reported in panel (a) applies to panels (a,c,d). Some anisotropic patterns have been enlarged by quoted scale factors relative to the first pattern (from the left) in panel (c).

**Figure 3:**
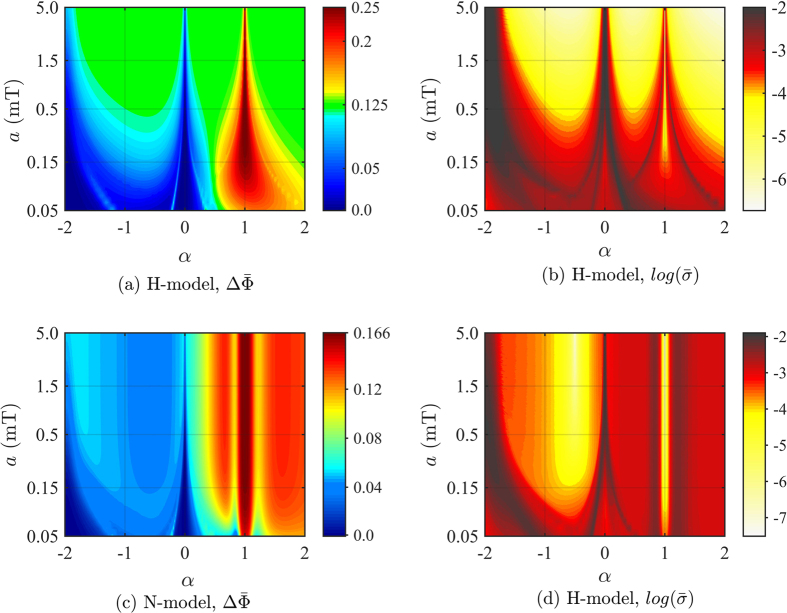
One-HFI radical pair models in an inhomogeneous ensemble of radical pairs with varying hyperfine interactions. H-model, where one electron spin is coupled to a spin-1/2 nucleus. N-model, where one electron spin is coupled to a spin-1 nucleus. Average signal strength 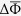 as a function of the average hyperfine strength *a* and axiality *α* for (**a**) the H-model and (**c**) the N-model. In both models three regions can be identified. The optimal region (*α* = 1) with an average signal strength of 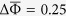 for the H-model and 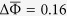 for the N-model. A plateau region with an average signal strength of 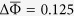 for the H-model and 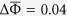 for the N-model, and the remain region where the signal strength depends on the axiality values. Signal uncertainty 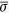, in logarithmic scale, as a function of *a* and *α* for (**b**) the H-model and (**d**) the N-model. For both models, in the optimal and plateau regions the signal uncertainty is greatly suppressed, leading to a more robust compass system. The Gaussian distribution of the hyperfine tensors has a percentage-based standard deviation of 2%. The lifetime of the radical pair is *τ* = 10 *μ*s and the strength of the external magnetic field is *B* = 0.05 mT. In the case of a constant-based standard deviation of e.g. 0.06 mT, the signal strength of the optimal region, for both models, decreases and the signal uncertainty significantly increases, thus leading to a loss of robustness, as described in the next section, and reported in Table 1.

**Figure 4:**
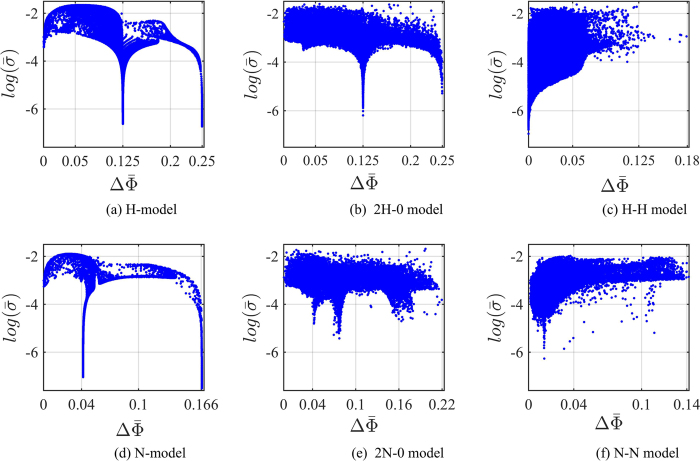
Signal uncertainty 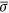, in logarithmic scale, vs. the average signal strength 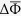 for (**a**) the H-model, where one radical has one hyperfine interaction with a spin-1/2 nucleus, (**b**) the 2H-0 model, where one radical has two hyperfine interactions with spin-1/2 nuclei, (**c**) the H-H model, where each radical has one hyperfine interaction with a spin-1/2 nucleus, (**d**) the N-model, where one radical has one hyperfine interaction with a spin-1 nucleus, (**e**) the 2N-0 model, where one radical has two hyperfine interactions with spin-1 nuclei and (**f**) the N-N model, where each radical has one hyperfine interaction with a spin-1 nucleus. In (**a,d**) 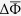 and 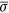 are the values used in Fig. 3. In (**b,c,e,f**) 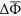 and 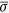 are calculated over 10^5^ samples of inhomogeneous nuclear spin environments as described in the text. A percentage-based standard deviation of 2% is used for all models. The lifetime of the radical pair is set to *τ* = 10 *μ*s and the strength of the external magnetic field is set as geomagnetic field *B* = 0.05 mT. By adding a second hyperfine interaction to the one-HFI models, signal uncertainty and signal strength change depending on the arrangement and type of nuclei of the two hyperfine interactions.

**Figure 5:**
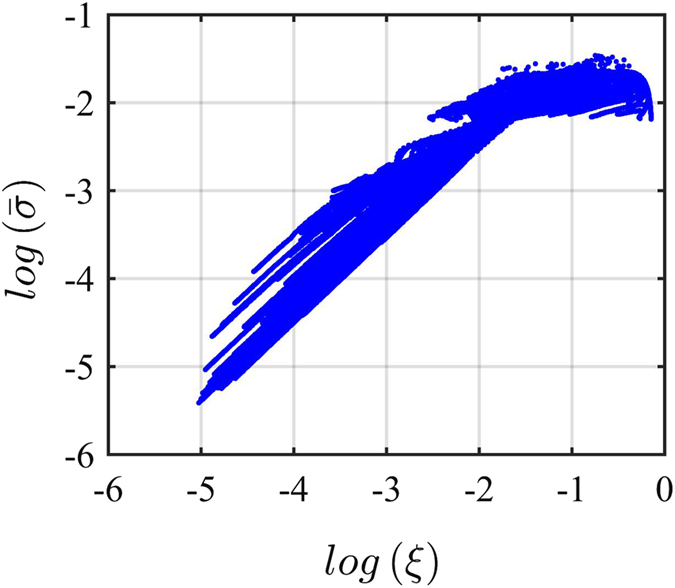
Signal uncertainty 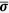 vs. *ξ*, i.e. the deviation of the average singlet yield 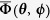 from the singlet yield of the homogeneous ensemble Φ^*o*^(*θ*, Φ). The values of 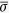 and *ξ* are both in logarithmic scale. There is a correlation between 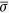 and *ξ*, with both decreasing or increasing. This indicates that for a non-robust compass system, in which the noise leads to a large signal uncertainty, the average pattern of an inhomogeneous ensemble is very different from the pattern of the corresponding homogeneous ensemble (see Fig. 2). Conversely, for a robust system average and homogeneous patterns are very similar. The model used is the H-model (see Fig. 3(b)). We find the same trend for all models studied here.

**Figure 6:**
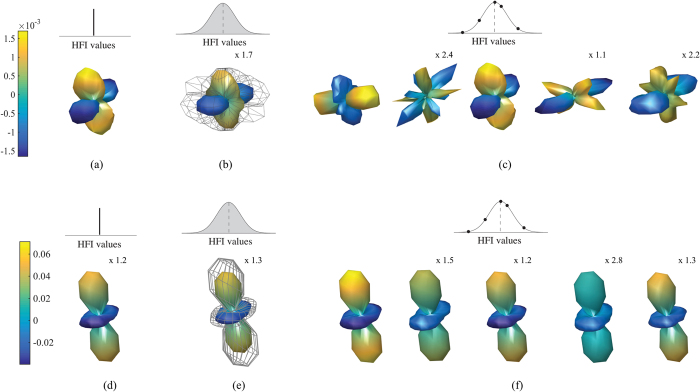
Effects of the inhomogeneous noise on two types of eight-nuclear spin systems with hyperfine values obtained from DFT calculations for cryptochrome. (top) FADH^•^-Trp: Radical pair system which comprises one radical with two nitrogen nuclei (N5, N10) taken from the flavin radical (FADH^•^) and the other radical with six hydrogen nuclei (H1, H2, H4, H6, H7) taken from the tryptophan radical (Trp). (bottom) FADH^•^-0: Radical pair system which comprises two nitrogen and six hydrogen nuclei (N5, N10, H5, H8*α*, H8*α*, H1′, H1′) in one radical taken from FADH^•^, while the other radical is devoid of hyperfine interactions. Panels (a,d) depict the anisotropic response of the homogeneous ensemble *Λ*^*o*^. Panels (b,e) depict the average anisotropic response of the inhomogeneous 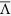, obtained by averaging over *N* anisotropic singlet yields Φ^*n*^. The grey grid represents the signal uncertainty, whose average resulted for (top) FADH^•^-Trp 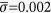 and for (bottom) FADH^•^-0 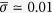. FADH^•^-Trp type is more robust to inhomogeneous noise than the FADH^•^-0 type. Panels (c,f) report the anisotropic patterns *Λ*^*n*^ for specific hyperfine parameters of different radical pairs of the inhomogeneous ensemble. The signal strength of FADH^•^-Trp resulted 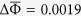 and that of FADH^•^-0 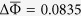. Thus the signal-to-noise ratio of FADH^•^-0 is larger of about ten fold than that of FADH^•^-Trp. For both models simulations were performed by setting the lifetime to *τ* = 1 *μ*s and the external magnetic field as geomagnetic field *B* = 0.05 mT. The number of samples of normally distributed hyperfine interactions was *N* = 3000, with a constant-based standard deviation of 0.06 mT. The color bar reported in panel (a) applies to panels (a–c), and the one reported in panel (d) applies to (**d–f**). Some anisotropic patterns have been enlarged by quoted scale factors relative to (top) the pattern in panel (a), and (bottom) the first individual pattern in panel (f). Hyperfine values are taken from the literature^30^.

As suggested in Fig. 1, different dimerization states, different states due to rearrangements of the C-terminus of cryptochrome for binding signaling proteins or due to metabolites binding, may lead to significantly larger variations of hyperfine couplings, greatly exacerbating the noise in an inhomogeneous ensemble. Of course, it may be possible that nature avoids such additional heterogeneities by favoring one form (e.g. the monomer form over the dimer form) for the design of functional magnetoreceptor ensembles. It might seem intuitive that evolution would favor such ensembles over ensembles involving multiple receptor states. However, a counterargument is that such differential states open up the possibility of adding potentially desirable control elements, such as feedback loops. This can be seen in another example of a receptor system operating near physical detection limits, bacterial chemotaxis. Instead of avoiding variation, nature here purposely varies the signal strength of its receptors by methylation to establish a negative feedback loop critical for the detection of weak chemical gradients^77^. This example is not unique, but phosphorylation and methylation, i.e. two processes that lead to receptor states with differing sensitivity provide in many signaling systems the key feedback step to make a receptor system work^24^. Given that little is known about how a potential magnetosensitive step in the cryptochrome photocycle is embedded in biochemical regulatory framework, it appears premature to dismiss the possibility that regulation favors an ensemble with multiple receptor states. Beyond phosphorylation, a process occurring in cryptochromes, such different regulatory states could be realized also through different levels of metabolite binding, such as ATP. If such multiple receptor states exist in an ensemble, it is possible that the size of variations of hyperfine couplings is increased further from the level considered in our calculations. The observation of robust ensembles, put forth here, would provide a possible way for nature to avoid the conundrum of either having to favor a reduction of variations or of favoring the existence of additional regulatory states; it may be possible to realize both.

One might argue that the signal uncertainty does not pose a serious obstacle because using a larger ensemble would allow to reduce this noise by averaging. Indeed, one expects the noise to be reduced by a factor of 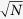, where N is the number of copies of sensory molecules involved in producing a magnetic signal. However, one needs to recognize that at any given time only a small subset of sensory molecules is actively involved in signaling. For cryptochromes, with a molar extinction coefficient on the order of *ε* = 10^4^ M^−1^ cm^−1^ and irradiation levels as used in bird magnetic orientation experiments, one can estimate that out of one million copies of cryptochromes in an outer segment of a photoreceptor cell, fewer than 100 would be activated by light every second. Reducing noise by averaging would thus either come at a high metabolic cost and ultimately be limited by the number of cryptochromes that could be expressed in a cell or would require averaging over longer times, thus slowing down the speed of the magnetic sensory system. Both of these undesirable effects could be avoided by selecting a sensory molecule with a small signal uncertainty. In a different sensory modality, vision, the remarkable sensitivity to a single photon is only possible because the sensory biomolecule rhodopsin has an extremely low probability of spontaneous isomerization^78^, i.e. an extremely low background noise level.

If the variations arise through thermal fluctuations, a certain degree of averaging will occur due to fluctuations on a time scale faster compared to the radical pair reaction times. Our treatment does not calculate the effect of such averaging. Such calculations are computationally expensive and are very uncertain without knowledge of the fluctuation sizes over different time scales. Our current study is only a first step into the investigation of noise through radical pair parameter variations. Instead of using Gaussian distributions to model variations, one would ideally evaluate the effect of particular conformational changes or aminoacid substitutions of a protein-radical pair complex on the hyperfine interactions. Combining such calculations with more detailed information would provide a better description of the variations in the radical pair ensembles and may allow one to arrive at experimentally testable predictions.

There are other sources of noises that need to be considered for a full analysis, such as molecular shot noise in later signal transduction steps^33^, noise arising from imperfect geometrical ordering of radical pairs^22^,^28^,^29^, and spin relaxation mechanisms^38^. Unlike other sources of noise, spin relaxation mechanisms and hyperfine inhomogeneity stem from protein motions. While relaxation processes stem from protein motions that are on time scales shorter than spin-correlation times, hyperfine inhomogeneity, discussed here, is realized from protein motions that occur at time scales larger than spin-correlation times. It would certainly be worth investigating whether the librational motion found to increase compass sensitivity^38^,^79^ could occur in a protein that is also robust against hyperfine inhomogeneity or whether nature would have to “choose” between the two and, if so, which one would provide the greater benefit for compass magnetoreception.

Other interesting scenario can emerge by investigating possible realizations of hyperfine inhomogeneity in a biological environment. For example, it was shown that substrate binding affects the geometry of the flavin ring^80^, thereby raising the question whether different conformational states of the protein-cofactor complex would lead to different motional constraints of the flavin ring, realizing in this way inhomogeneity on short time scales.

If evolution over millions of years has optimized radical-pair-based magnetic compasses, what did it act on? Were ways found to better fix molecules in ordered geometrical arrangements? Was the signal transduction network improved to amplify the initial small signals from the primary sensors? Or was the biomolecule for the primary response improved to yield a maximal signal, possibly through evolving motions causing spin relaxation? Here, we add a new idea to the discussion: selection may find biomolecules that are particularly good in preventing the primary signal from deteriorating because of the inhomogeneity present in a biological environment.

## Additional Information

**How to cite this article**: Procopio, M. and Ritz, T. Inhomogeneous ensembles of radical pairs in chemical compasses. *Sci. Rep.* **6**, 35443; doi: 10.1038/srep35443 (2016).

**Publisher’s note:** Springer Nature remains neutral with regard to jurisdictional claims in published maps and institutional affiliations.

## Figures and Tables

**Table 1:** One-HFI radical pair models with different percentage-based (2%, 5% and 10%) and fixed-width (0.02, 0.06 and 0.1 mT) standard deviations Δ of normally distibuted hyperfine variations.

Δ	Optimal			Plateau			Non-robust		
	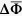	log 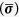	S/N	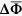	log 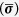	S/N	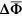	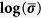	S/N
(a) H-model									
2%	0.25	−6.7	1.4 10^6^	0.125	−6.5	3.7 10^5^	0.11	−2.1	14.0
5%	0.25	−6.3	5.3 10^5^	0.125	−6.0	1.4 10^5^	0.077	−1.8	4.5
10%	0.25	−6.0	2.5 10^5^	0.125	−5.7	6.6 10^4^	0.076	−1.6	3.0
0.02	0.236	−2.0	26.0	0.125	−7.1	1.7 10^6^	0.07	−1.5	2.0
0.06	0.20	−1.7	10.0	0.125	−6.7	5.7 10^5^	0.06	−1.3	1.3
0.1	0.18	−1.7	9.0	0.125	−6.4	3.3 10^5^	0.06	−1.3	1.3
(b) N-model									
2%	0.1666	−7.5	4.7 10^6^	0.04	−7.0	4.4 10^5^	0.06	−2.0	6.0
5%	0.1666	−7.1	2.0 10^6^	0.04	−6.6	1.7 10^5^	0.04	−1.9	3.0
10%	0.1666	−6.8	1.0 10^6^	0.04	−6.3	8.0 10^4^	0.03	−1.8	2.0
0.02	0.1666	−5.3	3.6 10^4^	0.04	−6.9	3.0 10^5^	0.02	−1.5	0.65
0.06	0.1666	−4.1	2.3 10^3^	0.04	−6.0	3.5 10^4^	0.03	−1.2	0.5
0.1	0.1665	−3.5	4.7 10^2^	0.04	−5.5	1.2 10^4^	0.04	−1.2	0.67

Signal strength 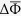, robustness in logarithm scale 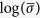 and signal-to-noise ratio S/N of selected radical pairs from the optimal, plateau and least robust regions, for (a) the H-model and (b) the N-model. For both models, the selected radical pair from the optimal region has hyperfine strength *a* = 5.0 mT and axiality *α* = 1.0, from the plateau region has strength *a* = 5.0 mT, and axiality *α* = 0.5 for the H-model and *α* = −0.5 for the N-model, from the least robust region has strength *a* = 0.05 mT, and *α* = 0.4 for the H-model and *α* = −0.22 for the N-model (see Fig. 3). In all calculations the lifetime is *τ* = 10 *μ*s, and the geomagnetic field is set to *B* = 0.05 mT. The signal-no-noise ratio is obtained considering 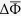 as the signal S, and 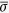 as the noise N.

**Table 2:** Two-HFIs radical pair models with different percentage-based (2%, 5% and 10%) and fixed-width (0.02, 0.06 and 0.1 mT) standard deviations Δ of normally distributed hyperfine variations.

Δ	Optimal			Plateau			Non-robust		
	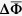	log 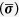	S/N	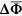	log 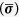	S/N	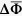	log 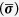	S/N
(a) 2H-0 model									
2%	0.25	−5.4	6.1 10^4^	0.125	−6.3	2.3 10^5^	0.211	−1.4	4.8
5%	0.25	−4.8	1.5 10^4^	0.125	−5.8	8.9 10^4^	0.206	−1.3	4.3
10%	0.25	−4.3	5.0 10^3^	0.125	−5.5	4.3 10^4^	0.212	−1.4	5.0
0.02	0.25	−5.1	3.7 10^4^	0.125	−5.7	5.8 10^4^	0.218	−2.0	55.0
0.06	0.25	−4.4	6.8 10^3^	0.125	−5.0	1.3 10^4^	0.217	−1.9	16.0
0.1	0.25	−4.0	2.7 10^3^	0.125	−4.7	6.2 10^3^	0.216	−1.5	7.2
(b) 2N-0 model									
2%	0.21	−4.3	4.1 10^3^	0.04	−4.7	2.3 10^3^	0.197	−1.7	9.9
5%	0.21	−3.6	8.0 10^2^	0.04	−4.0	4.0 10^2^	0.197	−1.68	9.5
10%	0.21	−3.2	3.7 10^2^	0.04	−3.7	1.8 10^2^	0.198	−1.8	12.0
0.02	0.21	−4.1	2.5 10^3^	0.04	−4.4	1.0 10^3^	0.198	−2.3	39.6
0.06	0.21	−3.4	4.8 10^2^	0.04	−3.9	3.4 10^2^	0.198	−2.2	32.0
0.1	0.21	−3.2	3.7 10^2^	0.04	−3.7	1.9 10^2^	0.198	−1.8	12.0

Signal strength 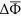, robustness in logarithm scale 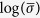 and signal-to-noise ratio S/N of selected radical pairs from the optimal, plateau and least robust regions, for (a) the 2H-0 model and (b) the 2N-0 model. (a) 2H-0 model. The selected radical pair from the optimal region has hyperfine strengths *a*_1_ = 2.2 mT, *a*_2_ = 3.5 mT, axiality *α*_1_ = 0.98, *α*_2_ = 1.0, and angle between the two hyperfine interactions *ψ* = 0.0. The selected radical pair from the plateau region has *a*_1_ = 4.6 mT, *a*_2_ = 0.06 mT, *α*_1_ = 1.84, *α*_2_ = −0.4, and *ψ* = 18.0. The selected radical pair from the least robust region has, *a*_1_ = 3.7 mT, *a*_2_ = 1.8 mT, *α*_1_ = 0.7, *α*_2_ = 1.7, and *ψ* = 0.0. (b) N-model. The selected radical pair from the optimal region has hyperfine strengths *a*_1_ = 1.0 mT, *a*_2_ = 4.3 mT, axiality *α*_1_ = 0.97, *α*_2_ = 1.0, and *ψ* = 1.0. The selected radical pair from the plateau region has *a*_1_ = 3.1 mT, *a*_2_ = 0.3 mT, *α*_1_ = −0.7, *α*_2_ = 0.7, and *ψ* = 76.0. The selected radical pair from the least robust region of the 2N-0 mdoel has same parameters as the one selected from the least robust 2H-0 model. In all calculations the lifetime is *τ* = 10 *μ*s, and the geomagnetic field is set to *B* = 0.05 mT. The signal-no-noise ratio is obtained considering 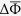 as the signal S, and 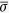 as the noise N.

**Table 3:** Multi-nuclear radical pair systems.

Δ	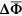	log 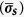	S/N
(a) FADH-Tr			
2%	0.0028	−3.5	8.5
5%	0.0025	−3.2	3.8
10%	0.0022	−3.0	2.4
0.2	0.0028	−3.2	3.9
0.6	0.0020	−2.8	1.0
1	0.0016	−2.6	0.6
(b) FADH-0			
2%	0.086	−3.1	99.0
5%	0.085	−2.6	37.0
10%	0.084	−2.4	19.0
0.2	0.084	−2.6	35.0
0.6	0.083	−2.1	11.6
1	0.075	−2.0	8.9

Signal strength 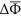, signal uncertainty 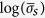 and signal-to-noise ratio S/N for different percentage-based (2%, 5%, 10%) and fixed-width (0.02, 0.06, 0.1 mT) standard deviation Δ of hyperfine variations, of (a) the FADH-Tr model, and (b) the FADH-0 model. The signal strength is 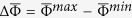. The signal uncertainty 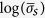 is calculated by averaging the signal uncertainty of 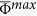 and 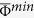. The signal-to-noise ratio S/N is calculated considering 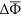 being the signal S, and 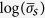 being the noise N. The lifetime is *τ* = 1 *μ*s, and the geomagnetic field is set to *B* = 0.05 mT.

## References

[b1] Wiltschko R. & Wiltschko W. Magnetic Orientation in Animals (Springer Verlag, Berlin Heidelberg, New York, 1995).

[b2] Wiltschko R. & Wiltschko W. Magnetoreception. BioEssays 28, 157–168 (2006). http://dx.doi.org/10.1002/bies.20363. 16435299 10.1002/bies.20363

[b3] Kirschvink J. L. & Gould J. L. Biogenic magnetite as a basis for magnetic field detection in animals. Biosystems 13, 181–201 (1981). http://www.sciencedirect.com/science/article/pii/0303264781900605. 7213948 10.1016/0303-2647(81)90060-5

[b4] Wiltschko R. & Wiltschko W. The magnetite-based receptors in the beak of birds and their role in avian navigation. Journal of Comparative Physiology A 199, 89–98 (2013). http://dx.doi.org/10.1007/s00359-012-0769-3. 10.1007/s00359-012-0769-3 PMC3552369 23111859

[b5] Schulten K., Swenberg C. E. & Weller A. A biomagnetic sensory mechanism based on magnetic field modulated coherent electron spin motion. Zeitschrift für Physikalische Chemie 111, 1–5 (1978). http://www.ks.uiuc.edu/Publications/Papers/PDF/SCHU78C/SCHU78C.pdf.

[b6] Schulten K. Magnetic field effects in chemistry and biology. Festkörperprobleme 22, 61–63 (1982). http://www.ks.uiuc.edu/Publications/Papers/paper.cgi?tbcode=SCHU82.

[b7] Ritz T., Adem S. & Schulten K. A model for Photoreceptor-Based magnetoreception in birds. Biophysical Journal 78, 707–718 (2000). http://www.cell.com/biophysj/abstract/S0006-3495(00)76629-X. 10653784 10.1016/S0006-3495(00)76629-X PMC1300674

[b8] Lohmann K. J. Q&a: Animal behaviour: Magnetic-field perception. Nature 464, 1140–1142 (2010). http://dx.doi.org/10.1038/4641140a. 20414302 10.1038/4641140a

[b9] Mouritsen H. & Hore P. The magnetic retina: light-dependent and trigeminal magnetoreception in migratory birds. Current Opinion in Neurobiology 22, 343–352 (2012). http://www.sciencedirect.com/science/article/pii/S0959438812000074. 22465538 10.1016/j.conb.2012.01.005

[b10] Stoneham A. M., Gauger E. M., Porfyrakis K., Benjamin S. C. & Lovett B. W. A new type of radical-pair-based model for magnetoreception. Biophys. J. 102, 961–968 (2012). http://www.sciencedirect.com/science/article/pii/S0006349512000616. 22404918 10.1016/j.bpj.2012.01.007 PMC3296028

[b11] Ritz T., Thalau P., Phillips J. B., Wiltschko R. & Wiltschko W. Resonance effects indicate a radical-pair mechanism for avian magnetic compass. Nature 429, 177–180 (2004). http://dx.doi.org/10.1038/nature02534. 15141211 10.1038/nature02534

[b12] Maeda K. . Chemical compass model of avian magnetoreception. Nature 453, 387–390 (2008). http://dx.doi.org/10.1038/nature06834. 18449197 10.1038/nature06834

[b13] Mouritsen H. . Cryptochromes and neuronal-activity markers colocalize in the retina of migratory birds during magnetic orientation. Proc. Nat. Acad. Sci. USA 101, 14294–14299 (2004). http://www.pnas.org/content/101/39/14294.abstract. 15381765 10.1073/pnas.0405968101 PMC521149

[b14] Möller A., Sagasser S., Wiltschko W. & Schierwater B. Retinal cryptochrome in a migratory passerine bird: a possible transducer for the avian magnetic compass. Naturwissenschaften 91, 585–588 (2004). http://dx.doi.org/10.1007/s00114-004-0578-9. 15551029 10.1007/s00114-004-0578-9

[b15] Nießner C. . Avian ultraviolet/violet cones identified as probable magnetoreceptors. PLoS ONE 6, e20091 (2011). http://dx.doi.org/10.1371. 21647441 10.1371/journal.pone.0020091 PMC3102070

[b16] Ahmad M., Galland P., Ritz T., Wiltschko R. & Wiltschko W. Magnetic intensity affects cryptochrome-dependent responses in arabidopsis thaliana. Planta 225, 615–624 (2007). http://dx.doi.org/10.1007/s00425-006-0383-0. 16955271 10.1007/s00425-006-0383-0

[b17] Gegear R. J., Casselman A., Waddell S. & Reppert S. M. Cryptochrome mediates light-dependent magnetosensitivity in drosophila. Nature 454, 1014–1018 (2008). http://dx.doi.org/10.1038/nature07183. 18641630 10.1038/nature07183 PMC2559964

[b18] Yoshii T., Ahmad M. & Helfrich-Förster C. Cryptochrome mediates light-dependent magnetosensitivity of *drosophila’s* circadian clock. PLoS Biol 7, e1000086 (2009). http://www.plosbiology.org/article/info:doi/10.1371/journal.pbio.1000086. 19355790 10.1371/journal.pbio.1000086 PMC2667543

[b19] Marley R., Giachello C. N. G., Scrutton N. S., Baines R. A. & Jones A. R. Cryptochrome-dependent magnetic field effect on seizure response in drosophila larvae. Scientific Reports 4, – (2014). http://dx.doi.org/10.1038/srep05799. 10.1038/srep05799 PMC4107376 25052424

[b20] Maeda K. . Magnetically sensitive light-induced reactions in cryptochrome are consistent with its proposed role as a magnetoreceptor. Proc. Nat. Acad. Sci. USA 109, 4774–4779 (2012). http://www.pnas.org/content/109/13/4774.abstract. 22421133 10.1073/pnas.1118959109 PMC3323948

[b21] Neil S. R. T. . Broadband cavity-enhanced detection of magnetic field effects in chemical models of a cryptochrome magnetoreceptor. The Journal of Physical Chemistry B 118, 4177–4184 (2014). http://dx.doi.org/10.1021/jp500732u. 24655160 10.1021/jp500732u

[b22] Solov’yov I. A., Mouritsen H. & Schulten K. Acuity of a cryptochrome and vision-based magnetoreception system in birds. Biophysics Journal 99, 40–49 (2010). http://www.sciencedirect.com/science/article/pii/S0006349510004194. 10.1016/j.bpj.2010.03.053 PMC2895366 20655831

[b23] Rodgers C. T. Magnetic field effects in chemical systems. Pure Appl. Chem. 81, 19–43 (2009). http://dx.doi.org/10.1351/PAC-CON-08-10-18.

[b24] Chaves I. . The cryptochromes: blue light photoreceptors in plants and animals. Annu Rev Plant Biol 62, 335–364 (2011). http://www.ncbi.nlm.nih.gov/pubmed/21526969. 21526969 10.1146/annurev-arplant-042110-103759

[b25] Timmel C., Cintolesi F., Brocklehurst B. & Hore P. Model calculations of magnetic field effects on the recombination reactions of radicals with anisotropic hyperfine interactions. Chem. Phys. Lett. 334, 387–395 (2001). http://www.sciencedirect.com/science/article/pii/S0009261400014366.

[b26] Cintolesi F., Ritz T., Kay C., Timmel C. & Hore P. Anisotropic recombination of an immobilized photoinduced radical pair in a magnetic field: a model avian photomagnetoreceptor. Chem. Phys. 294, 385–399 (2003). http://www.sciencedirect.com/science/article/pii/S0301010403003203.

[b27] Efimova O. & Hore P. Role of exchange and dipolar interactions in the radical pair model of the avian magnetic compass. Biophys. J. 94, 1565–1574 (2008). http://www.sciencedirect.com/science/article/pii/S0006349508705952. 17981903 10.1529/biophysj.107.119362 PMC2242753

[b28] Hill E. & Ritz T. Can disordered radical pair systems provide a basis for a magnetic compass in animals? J. Royal Soc. Interface (2009). http://rsif.royalsocietypublishing.org/content/early/2009/11/10/rsif.2009.0378.focus.abstract. 10.1098/rsif.2009.0378.focus PMC2844000 19906676

[b29] Lau J. C. S., Wagner-Rundell N., Rodgers C. T., Green N. J. B. & Hore P. J. Effects of disorder and motion in a radical pair magnetoreceptor. Journal of The Royal Society Interface (2009). http://rsif.royalsocietypublishing.org/content/early/2009/12/07/rsif.2009.0399.focus. 10.1098/rsif.2009.0399.focus PMC2844003 20007172

[b30] Lau J. C. S., Rodgers C. T. & Hore P. J. Compass magnetoreception in birds arising from photo-induced radical pairs in rotationally disordered cryptochromes. J. Royal Soc. Interface 9, 3329–3337 (2012). http://rsif.royalsocietypublishing.org/content/9/77/3329.abstract. 10.1098/rsif.2012.0374 PMC3481564 22977104

[b31] Nießner C. . Magnetoreception: activated cryptochrome 1a concurs with magnetic orientation in birds. Journal of The Royal Society Interface 10 (2013). http://rsif.royalsocietypublishing.org/content/10/88/20130638. 10.1098/rsif.2013.0638 PMC3785833 23966619

[b32] Bolte P. . Localisation of the putative magnetoreceptive protein cryptochrome 1b in the retinae of migratory birds and homing pigeons. PLoS ONE 11, e0147819 (2016). http://www.ncbi.nlm.nih.gov/pmc/articles/PMC4783096/. 26953791 10.1371/journal.pone.0147819 PMC4783096

[b33] Weaver J. C., Vaughan T. E. & Astumian R. D. Biological sensing of small field differences by magnetically sensitive chemical reactions. Nature 405, 707–709 (2000). http://dx.doi.org/10.1038/35015128. 10864331 10.1038/35015128

[b34] Ritz T. . Magnetic compass of birds is based on a molecule with optimal directional sensitivity. Biophysical Journal 96, 3451–3457 (2009). http://www.sciencedirect.com/science/article/pii/S0006349509004688. 19383488 10.1016/j.bpj.2008.11.072 PMC2718301

[b35] Cai J. Quantum probe and design for a chemical compass with magnetic nanostructures. Phys. Rev. Lett. 106, 100501 (2011). http://link.aps.org/doi/10.1103/PhysRevLett.106.100501. 21469779 10.1103/PhysRevLett.106.100501

[b36] Cai J., Caruso F. & Plenio M. B. Quantum limits for the magnetic sensitivity of a chemical compass. Phys. Rev. A 85, 040304 (2012). http://link.aps.org/doi/10.1103/PhysRevA.85.040304.

[b37] Lee A. A. . Alternative radical pairs for cryptochrome-based magnetoreception. Journal of The Royal Society Interface 11 (2014). http://rsif.royalsocietypublishing.org/content/11/95/20131063.abstract. 10.1098/rsif.2013.1063 PMC4006233 24671932

[b38] Kattnig D. R., Solov’yov I. A. & Hore P. J. Electron spin relaxation in cryptochrome-based magnetoreception. Phys. Chem. Chem. Phys. – (2016). http://dx.doi.org/10.1039/C5CP06731F. 10.1039/c5cp06731f 27020113

[b39] Gauger E. M., Rieper E., Morton J. J. L., Benjamin S. C. & Vedral V. Sustained quantum coherence and entanglement in the avian compass. Phys. Rev. Lett. 106, 040503 (2011). http://link.aps.org/doi/10.1103/PhysRevLett.106.040503. 21405313 10.1103/PhysRevLett.106.040503

[b40] Bandyopadhyay J. N., Paterek T. & Kaszlikowski D. Quantum coherence and sensitivity of avian magnetoreception. Phys. Rev. Lett. 109, 110502 (2012). http://link.aps.org/doi/10.1103/PhysRevLett.109.110502. 23005606 10.1103/PhysRevLett.109.110502

[b41] Frauenfelder H. & McMahon B. Energy landscape and fluctuations in proteins. Annalen der Physik 9, 655–667 (2000). http://dx.doi.org/10.1002/1521-3889(200010)9:9/10655::AID-ANDP6553.0.CO;2-Z.

[b42] Hofmann C., Aartsma T. J., Michel H. & Kohler J. Direct observation of tiers in the energy landscape of a chromoprotein: A single-molecule study. Proc. Nat. Acad. Sci. USA 100, 15534–15538 (2003). http://www.pnas.org/content/100/26/15534.abstract. 14671325 10.1073/pnas.2533896100 PMC307602

[b43] Pauwels E. . Influence of protein environment on the electron paramagnetic resonance properties of flavoprotein radicals: A qm/mm study. The Journal of Physical Chemistry B 114, 16655–16665 (2010). http://pubs.acs.org/doi/abs/10.1021/jp109763t. 21090702 10.1021/jp109763t

[b44] Sang Y. . N-terminal domain–mediated homodimerization is required for photoreceptor activity of arabidopsis cryptochrome 1. The Plant Cell Online 17, 1569–1584 (2005). http://www.plantcell.org/content/17/5/1569.abstract. 10.1105/tpc.104.029645 PMC1091775 15805487

[b45] Klar T., Pokorny R., Moldt J., Batschauer A. & Essen L.-O. Cryptochrome 3 from arabidopsis thaliana: Structural and functional analysis of its complex with a folate light antenna. Journal of Molecular Biology 366, 954–964 (2007). http://www.sciencedirect.com/science/article/pii/S0022283606016251. 17188299 10.1016/j.jmb.2006.11.066

[b46] Partch C. L. & Sancar A. Photochemistry and photobiology of cryptochrome blue-light photopigments: The search for a photocycle. Photochemistry and Photobiology 81, 1291–1304 (2005). http://dx.doi.org/10.1562/2005-07-08-IR-607. 16164372 10.1562/2005-07-08-IR-607

[b47] Partch C. L., Clarkson M. W., Özgür S., Lee A. L. & Sancar A. Role of structural plasticity in signal transduction by the cryptochrome blue-light photoreceptor. Biochemistry 44, 3795–3805 (2005). http://dx.doi.org/10.1021/bi047545g. 15751956 10.1021/bi047545g

[b48] Kondoh M. . Light-induced conformational changes in full-length arabidopsis thaliana cryptochrome. Journal of Molecular Biology 413, 128–137 (2011). http://www.sciencedirect.com/science/article/pii/S0022283611009296. 21875594 10.1016/j.jmb.2011.08.031 PMC4451184

[b49] Lian H.-L. . Blue-light-dependent interaction of cryptochrome 1 with spa1 defines a dynamic signaling mechanism. Genes & Development 25, 1023–1028 (2011). http://www.ncbi.nlm.nih.gov/pmc/articles/PMC3093117/. 21511872 10.1101/gad.2025111 PMC3093117

[b50] Burney S. . Conformational change induced by {ATP} binding correlates with enhanced biological function of arabidopsis cryptochrome. {FEBS} Letters 583, 1427–1433 (2009). http://www.sciencedirect.com/science/article/pii/S0014579309002294. 10.1016/j.febslet.2009.03.040 19327354

[b51] Engelhard C. . Cellular metabolites enhance the light sensitivity of arabidopsis cryptochrome through alternate electron transfer pathways. The Plant Cell Online (2014). http://www.plantcell.org/content/early/2014/11/21/tpc.114.129809.abstract. 10.1105/tpc.114.129809 PMC4277212 25428980

[b52] Müller P. . Atp binding turns plant cryptochrome into an efficient natural photoswitch. Scientific Reports 4 (2014). http://dx.doi.org/10.1038/srep05175. 10.1038/srep05175 PMC4046262 24898692

[b53] Medina M., Vrielink A. & Cammack R. Esr and electron nuclear double resonance characterization of the cholesterol oxidase from brevibacterium sterolicum in its semiquinone state. European Journal of Biochemistry 222, 941–947 (1994). http://dx.doi.org/10.1111/j.1432-1033.1994.tb18943.x. 8026504 10.1111/j.1432-1033.1994.tb18943.x

[b54] Medina M., Gomez-Moreno C. & Cammack R. Electron spin resonance and electron nuclear double resonance studies of flavoproteins involved in the photosynthetic electron transport in the cyanobacterium anabaena sp. pcc 7119. European Journal of Biochemistry 227, 529–536 (1995). http://dx.doi.org/10.1111/j.1432-1033.1995.tb20420.x. 7851433 10.1111/j.1432-1033.1995.tb20420.x

[b55] Medina M., Vrielink A. & Cammack R. Electron spin echo envelope modulation studies of the semiquinone anion radical of cholesterol oxidase from brevibacterium sterolicum. FEBS Letters 400, 247–251 (1997). http://dx.doi.org/10.1016/S0014-5793(96)01372-5. 9001407 10.1016/s0014-5793(96)01372-5

[b56] Weber S. . Substrate binding to {DNA} photolyase studied by electron paramagnetic resonance spectroscopy. Biophysical Journal 81, 1195–1204 (2001). http://www.sciencedirect.com/science/article/pii/S0006349501757773. 11463661 10.1016/S0006-3495(01)75777-3 PMC1301589

[b57] Weber S., Kay C. W. M., Bacher A., Richter G. & Bittl R. Probing the n(5) h bond of the isoalloxazine moiety of flavin radicals by x- and w-band pulsed electron–nuclear double resonance. ChemPhysChem 6, 292–299 (2005). http://dx.doi.org/10.1002/cphc.200400377. 15751352 10.1002/cphc.200400377

[b58] Medina M. . Electron-nuclear double resonance and hyperfine sublevel correlation spectroscopic studies of flavodoxin mutants from anabaena sp. {PCC} 7119. Biophysical Journal 77, 1712–1720 (1999). http://www.sciencedirect.com/science/article/pii/S0006349599770177. 10465780 10.1016/S0006-3495(99)77017-7 PMC1300457

[b59] Schleicher E. . The electronic state of flavoproteins: Investigations with proton electron–nuclear double resonance. Applied Magnetic Resonance 37, 339–352 (2010). http://dx.doi.org/10.1007/s00723-009-0101-8. 26089595 10.1007/s00723-009-0101-8 PMC4469238

[b60] Schleicher E. . Electron nuclear double resonance differentiates complementary roles for active site histidines in (6-4) photolyase. Journal of Biological Chemistry 282, 4738–4747 (2007). http://www.jbc.org/content/282/7/4738.abstract. 17164245 10.1074/jbc.M604734200

[b61] Brosi R., Bittl R. & Engelhard C. Flavins and Flavoproteins: Methods and Protocols (Springer New York, New York, NY, 2014). http://dx.doi.org/10.1007/978-1-4939-0452-5_13.

[b62] Weber S., Möbius K., Richter G. & Kay C. W. M. The electronic structure of the flavin cofactor in dna photolyase. Journal of the American Chemical Society 123, 3790–3798 (2001). http://dx.doi.org/10.1021/ja003426m. 11457111 10.1021/ja003426m

[b63] García J. I. . Theoretical analysis of the electron spin density distribution of the flavin semiquinone isoalloxazine ring within model protein environments. The Journal of Physical Chemistry A 106, 4729–4735 (2002). http://dx.doi.org/10.1021/jp014696+.

[b64] Steiner U. E. & Ulrich T. Magnetic field effects in chemical kinetics and related phenomena. Chem. Rev. 89, 51–147 (1989). http://pubs.acs.org/doi/abs/10.1021/cr00091a003.

[b65] Haberkorn R. Density matrix description of spin-selective radical pair reactions. Molecular Physics 32, 1491–1493 (1976). http://www.tandfonline.com/doi/abs/10.1080/00268977600102851.

[b66] Jones J. & Hore P. Spin-selective reactions of radical pairs act as quantum measurements. Chemical Physics Letters 488, 90–93 (2010). http://www.sciencedirect.com/science/article/pii/S000926141000120X.

[b67] Jones J., Maeda K., Steiner U. & Hore P. Reply to comment on ‘spin-selective reactions of radical pairs act as quantum measurements’. Chemical Physics Letters 508, 184–185 (2011). http://www.sciencedirect.com/science/article/pii/S0009261411004167.

[b68] Jones J., Maeda K. & Hore P. Reaction operators for spin-selective chemical reactions of radical pairs. Chemical Physics Letters 507, 269–273 (2011). http://www.sciencedirect.com/science/article/pii/S0009261411003666. 10.1063/1.4844355 24359369

[b69] Ivanov K. L., Petrova M. V., Lukzen N. N. & Maeda K. Consistent treatment of spin-selective recombination of a radical pair confirms the haberkorn approach. The Journal of Physical Chemistry A 114, 9447–9455 (2010). http://pubs.acs.org/doi/abs/10.1021/jp1048265. 20704353 10.1021/jp1048265

[b70] Shushin A. I. Effect of state-selective reactive decay on the evolution of quantum systems. The J. of Chem. Phys. 133, – (2010). http://scitation.aip.org/content/aip/journal/jcp/133/4/10.1063/1.3461133. 10.1063/1.3461133 20687661

[b71] Werner H., Schulten Z. & Schulten K. Theory of the magnetic field modulated geminate recombination of radical ion pairs in polar solvents: Application to the pyrene n,n dimethylaniline system. The Journal of Chemical Physics 67, 646–663 (1977). http://scitation.aip.org/content/aip/journal/jcp/67/2/10.1063/1.434868.

[b72] Kaptein R. & Oosterhoff J. Chemically induced dynamic nuclear polarization ii: (relation with anomalous {ESR} spectra). Chemical Physics Letters 4, 195–197 (1969). http://www.sciencedirect.com/science/article/pii/0009261469800989.

[b73] Timmel C., Till U., Brocklehurst B., Mclauchlan K. & Hore P. Effects of weak magnetic fields on free radical recombination reactions. Mol. Phys. 95, 71–89 (1998). http://www.tandfonline.com/doi/abs/10.1080/00268979809483134. 10.1080/09553000050176270 11098854

[b74] Brocklehurst B. Spin correlation in the geminate recombination of radical ions in hydrocarbons. part 1.-theory of the magnetic field effect. J. Chem. Soc. 2 72, 1869–1884 (1976). http://dx.doi.org/10.1039/F29767201869.

[b75] Brautigam C. A. . Structure of the photolyase-like domain of cryptochrome 1 from arabidopsis thaliana. Proceedings of the National Academy of Sciences of the United States of America 101, 12142–12147 (2004). http://www.ncbi.nlm.nih.gov/pmc/articles/PMC514401/. 15299148 10.1073/pnas.0404851101 PMC514401

[b76] Procopio M. *Radical-pair based compass magnetoreception.* Ph.D. thesis, University of Bologna (2011).

[b77] Yi T.-M., Huang Y., Simon M. I. & Doyle J. Robust perfect adaptation in bacterial chemotaxis through integral feedback control. Proceedings of the National Academy of Sciences of the United States of America 97, 4649–4653 (2000). http://www.ncbi.nlm.nih.gov/pmc/articles/PMC18287/. 10781070 10.1073/pnas.97.9.4649 PMC18287

[b78] Rieke F. & Baylor D. A. Single-photon detection by rod cells of the retina. Rev. Mod. Phys. 70, 1027–1036 (1998). http://link.aps.org/doi/10.1103/RevModPhys.70.1027.

[b79] Hiscock H. G. . The quantum needle of the avian magnetic compass. Proceedings of the National Academy of Sciences (2016). http://www.pnas.org/content/early/2016/03/30/1600341113.abstract. 10.1073/pnas.1600341113 PMC4855607 27044102

[b80] Lyubimov A. Y., Heard K., Tang H., Sampson N. S. & Vrielink A. Distortion of flavin geometry is linked to ligand binding in cholesterol oxidase. Protein Science: A Publication of the Protein Society 16, 2647–2656 (2007). http://www.ncbi.nlm.nih.gov/pmc/articles/PMC2222809/. 18029419 10.1110/ps.073168207 PMC2222809

